# New time scale generalizations of the Ostrowski-Grüss type inequality for *k* points

**DOI:** 10.1186/s13660-017-1525-6

**Published:** 2017-10-03

**Authors:** Eze R Nwaeze, Seth Kermausuor, Ana M Tameru

**Affiliations:** 1 Department of Mathematics, Tuskegee University, Tuskegee, AL 36088 USA; 2 Department of Mathematics and Computer Science, Alabama State University, Montgomery, AL 36104 USA

**Keywords:** 6D15, 54C30, 26D10, Montgomery identity, Grüss inequality, Ostrowski’s inequality, time scales, *k* points

## Abstract

Two Ostrowski-Grüss type inequalities for *k* points with a parameter $\lambda\in[0, 1]$ are hereby presented. The first generalizes a recent result due to Nwaeze and Tameru, and the second extends the result of Liu *et al*. to *k* points. Many new interesting inequalities can be derived as special cases of our results by considering different values of *λ* and $k\in\mathbb{N}$. In addition, we apply our results to the continuous, discrete, and quantum time scales to obtain several novel inequalities in this direction.

## Introduction

In 1997, Dragomir and Wang [3] (see also [4, 5] for related results) obtained the following inequality which is today known as the Ostrowski-Grüss inequality.

### Theorem 1

*If* $f:[a, b]\rightarrow\mathbb{R}$ *is differentiable on* $[a, b]$ *and* $\gamma\leq f'(x)\leq\Gamma$ *for all* $x\in[a, b]$ *for some constants* $\gamma, \Gamma\in\mathbb{R}$, *then*

1 $$ \bigg|f(x) - \frac{1}{b-a} \int_{a}^{b} f(t)\,\mathrm{d}t - \frac{f(b)-f(a)}{b-a} \biggl(x- \frac{a+b}{2} \biggr) \bigg|\leq\frac{1}{4}(b-a) (\Gamma- \gamma) $$

*for all* $x\in[a, b]$.

With the introduction of the theory of time scales (see Section 2), Tuna and Daghan [15] obtained the following time scale version of the Ostrowski-Grüss type inequality. Specifically, they proved the following.

### Theorem 2

*Let* $a, b, x, t \in\mathbb{T}$, $a< b$, *and* $f:[a, b]\rightarrow\mathbb {R}$ *be differentiable*. *If* $f^{\Delta}$ *is rd*-*continuous and* $\gamma \leq f^{\Delta}(t)\leq\Gamma$ *for all* $t\in[a, b]$ *and for some* $\gamma, \Gamma\in\mathbb{R}$, *then for all* $x\in[a, b]$, *we have*

2 $$ \begin{aligned}[b] & \bigg|f(x) - \frac{1}{b-a} \int_{a}^{b} f^{\sigma}(t)\Delta t - \frac {\Gamma+ \gamma}{2(b-a)} \bigl[h_{2}(x, a) - h_{2}(x, b) \bigr] \bigg| \\ &\quad\leq\frac{\Gamma- \gamma}{2(b-a)} \bigl[h_{2}(x, a) + h_{2}(x, b) \bigr]. \end{aligned}$$

Recently, Nwaeze and Tameru [14] proved the following generalization of Theorem 2 to *k* points.

### Theorem 3

*Suppose that*

1. $a, b\in\mathbb{T}$, $I_{k}:a=x_{0}< x_{1}<\cdots<x_{k-1}<x_{k}=b$ *is a partition of the interval* $[a, b]$ *for* $x_{0}, x_{1}, \ldots,x_{k}\in\mathbb{T}$;
2. $\alpha_{j}\in\mathbb{T}$ ($j=0,1,\ldots, k+1$) *is* $k+2$ *points so that* $\alpha_{0}=a$, $\alpha_{j}\in[x_{j-1}, x_{j}]$ ($j=1,\ldots, k$) *and* $\alpha _{k+1}=b$;
3. $f: [a, b]\rightarrow\mathbb{R}$ *is differentiable*, $f^{\Delta}$ *is rd*-*continuous*, *and there exist* $\gamma, \Gamma\in\mathbb{R}$ *such that* $\gamma\leq f^{\Delta}(t)\leq\Gamma$ *for all* $t\in[a, b]$.

*Then we have the following inequality*:

3 $$ \begin{aligned}[b] & \Bigg|\sum_{j=0}^{k} ( \alpha_{j+1}-\alpha_{j} )f(x_{j}) - \int _{a}^{b}f^{\sigma}(t)\Delta t - \frac{\Gamma+\gamma}{2} \sum_{j=0}^{k-1} \bigl(h_{2}(x_{j+1}, \alpha _{j+1}) -h_{2}(x_{j}, \alpha_{j+1}) \bigr) \Bigg| \\ &\quad\leq\frac{\Gamma-\gamma}{2} \sum_{j=0}^{k-1} \bigl(h_{2}(x_{j}, \alpha _{j+1}) + h_{2}(x_{j+1}, \alpha_{j+1}) \bigr). \end{aligned}$$

*Inequality* (3) *is sharp in the sense that the constant* 1/2 *on the right*-*hand side cannot be replaced by a smaller one*.

Many other variants of the Ostrowski-Grüss type inequality (on time scales) and related results (see, for example, [7–10, 12, 13] and the references therein) are bound in the literature. For the sake of this work, we present next a recent result due to Liu *et al*. [11].

### Theorem 4

*Let* $a, b, t, x\in\mathbb{T}$, $a< b$, *and* $f:[a, b]\rightarrow\mathbb {R}$ *be differentiable*. *Then*, *for all* $x\in[a, b]$, *we have*

4 $$ \begin{aligned}[b] & \bigg|f(x) - \frac{f(b)-f(a)}{b-a} \biggl(\frac{h_{2}(x, a)-h_{2}(x, b)}{b-a} \biggr) - \frac{1}{b-a} \int_{a}^{b} f^{\sigma}(t)\Delta t \bigg| \\ &\quad\leq \biggl[\frac{1}{b-a} \int_{a}^{b} S^{2}(x, t) \Delta t - \biggl( \frac {h_{2}(x, a)-h_{2}(x, b)}{b-a} \biggr)^{2} \biggr]^{\frac{1}{2}} \\ &\qquad{}\times \biggl[\frac{1}{b-a} \int_{a}^{b} \bigl(f^{\Delta}(t) \bigr)^{2} \Delta t - \biggl(\frac{f(b)-f(a)}{b-a} \biggr)^{2} \biggr]^{\frac{1}{2}}, \end{aligned}$$

*where*

$$ S(x, t)= \textstyle\begin{cases} t-a,& a\leq t< x,\\ t-b,& x\leq t\leq b. \end{cases} $$

The aim of this work is the following: generalize Theorem 3 via a parameter $\lambda\in[0, 1]$ such that for $\lambda=0$, we recover Theorem 3, and for $\lambda\in(0, 1]$ we get completely new results. Next, we extend Theorem 4 to *k* points.

This paper is organized as follows. In Section 2, we recall some definitions and results of the time scale theory. Thereafter, our results are stated and proved in Section 3. Finally, we apply our results to different time scales in Section 4.

## Time scale essentials

In order to unify the theory of integral and differential calculus with the calculus of finite difference, the German mathematician Stefan Hilger [6] in 1988 introduced the concept of time scales. We now present a brief overview of the theory of time scales. For an in-depth study, we invite the interested reader to see references [1, 2].

A *time scale* $\mathbb{T}$ is an arbitrary nonempty closed subset of $\mathbb{R}$. We assume throughout that a time scale $\mathbb{T}$ has the topology that it inherits from the real numbers with the standard topology. Since a time scale may not be connected, we need the following concept of jump operators.

The forward *jump operator* $\sigma:\mathbb{T}\rightarrow\mathbb {T}$ is defined by

$$\sigma(t):=\inf\{s\in\mathbb{T}: s>t\}, $$

while the backward *jump operator* $\rho:\mathbb{T}\rightarrow \mathbb{T}$ is defined by

$$\rho(t):=\sup\{s\in\mathbb{T}: s< t\}. $$

In this definition, we put $\inf\emptyset=\sup\mathbb{T}$ (i.e., $\sigma(t)=t$ if $\mathbb{T}$ has a maximum *t*) and $\sup\emptyset =\inf\mathbb{T}$ (i.e., $\rho(t)=t$ if $\mathbb{T}$ has a minimum *t*), where ∅ denotes the empty set. The jump operators *σ* and *ρ* allow the classification of points in $\mathbb{T}$ in this manner: if $\sigma(t)>t$, we say that *t* is *right-scattered*, while if $\rho(t)< t$ we say that *t* is *left-scattered*. Points that are right-scattered and left-scattered at the same time are called *isolated*. Also, if $t < \sup\mathbb{T}$ and $\sigma(t) = t$, then *t* is called right-dense, and if $t > \inf\mathbb{T}$ and $\rho(t) = t$, then *t* is called left-dense. Points that are right-dense and left-dense at the same time are called *dense*. We also introduce the sets $\mathbb{T}^{k}$, $\mathbb{T}_{k}$, and $\mathbb {T}^{k}_{k}$, which are derived from the time scale $\mathbb{T}$ as follows: if $\mathbb{T}$ has a left-scattered maximum $t_{1}$, then $\mathbb {T}^{k}=\mathbb{T}\setminus\{t_{1}\}$, otherwise $\mathbb{T}^{k}=\mathbb{T}$. If $\mathbb{T}$ has a right-scattered minimum $t_{2}$, then $\mathbb {T}_{k}=\mathbb{T}\setminus\{t_{2}\}$, otherwise $\mathbb{T}_{k}=\mathbb{T}$. Finally, we define $\mathbb{T}^{k}_{k}=\mathbb{T}^{k}\cap\mathbb{T}_{k}$.

For $a, b \in\mathbb{T}$ with $a\leq b$, we define the interval $[a, b]$ in $\mathbb{T}$ by $[a, b]=\{t\in\mathbb{T}: a\leq t\leq b\}$. Open intervals and half-open intervals are defined in the same manner.

### Definition 5

The function $f^{\sigma}:\mathbb{T}\rightarrow\mathbb{R}$ is defined as $f^{\sigma}(t)=f(\sigma(t))$.

### Definition 6

Delta derivative

Assume that $f:\mathbb{T\rightarrow R}$ is a function. Then the delta derivative $f^{\Delta}(t)\in\mathbb{R}$ at $t\in \mathbb{T}^{k}$ is defined to be number (provided it exists) with the property that, for any $\epsilon>0$, there exists a neighborhood *U* of *t* such that

$$ \bigl\vert f^{\sigma} (t)-f(s)-f^{\Delta}(t) \bigl[ \sigma(t)-s \bigr] \bigr\vert \leq\epsilon \bigl\vert \sigma(t)-s \bigr\vert ,\quad \forall s\in U. $$

If $\mathbb{T}=\mathbb{R}$, then $f^{\Delta}(t)=\frac{\mathrm{d}f(t)}{\mathrm{d}t}$, and if $\mathbb{T}=\mathbb{Z}$, then $f^{\Delta}(t)=f(t+1)-f(t)$.

### Theorem 7

*Let* $f, g:\mathbb{T}\rightarrow\mathbb{R}$ *be two differentiable functions at* $t\in{\mathbb{T}}^{k}$. *Then the product* $fg:\mathbb {T}\rightarrow\mathbb{R}$ *is also differentiable at* *t* *with*

$$(fg)^{\Delta}(t)=f^{\Delta}(t)g(t)+f^{\sigma}(t)g^{\Delta }(t)=f(t)g^{\Delta}(t)+f^{\Delta}(t)g^{\sigma}(t). $$

### Definition 8

The function $f:\mathbb{T}\rightarrow\mathbb{R}$ is said to be *rd*-continuous if it is continuous at all dense points $t\in\mathbb {T}$ and its left-sided limits exist at all left-dense points $t\in \mathbb{T}$.

### Definition 9

Let *f* be an *rd*-continuous function. Then $g:\mathbb{T}\rightarrow \mathbb{R}$ is called the antiderivative of *f* on $\mathbb{T}$ if it is differentiable on $\mathbb{T}$ and satisfies $g^{\Delta}(t)=f(t)$ for any $t\in{\mathbb{T}}^{k}$. In this case, we have

$$\int_{a}^{b} f(s)\Delta s = g(b)-g(a). $$

### Theorem 10

*If* $a,b,c \in\mathbb{T}$ *with* $a< c< b$, $\alpha\in\mathbb{R}$ *and* *f*, *g* *are* *rd*-*continuous*, *then*

(i) $\int_{a}^{b} [f(t)+g(t)]\Delta t=\int_{a}^{b} f(t)\Delta t + \int_{a}^{b} g(t)\Delta t$.
(ii) $\int_{a}^{b} \alpha f(t)\Delta t= \alpha\int_{a}^{b} f(t)\Delta t$.
(iii) $\int_{a}^{b} f(t)\Delta t= -\int_{b}^{a} f(t)\Delta t$.
(iv) $\int_{a}^{b} f(t)\Delta t=\int_{a}^{c} f(t)\Delta t + \int _{c}^{b} f(t)\Delta t$.
(v) $\vert \int_{a}^{b} f(t)\Delta t \vert \leq\int_{a}^{b} |f(t)|\Delta t$.
(vi) $\int_{a}^{b} f(t)g^{\Delta}(t)\Delta t=(fg)(b) - (fg)(a) - \int_{a}^{b} f^{\Delta}(t)g^{\sigma}(t)\Delta t$.

### Definition 11

Let $h_{k}:\mathbb{T}^{2}\rightarrow\mathbb{R}$, $k\in\mathbb{N}$ be functions that are recursively defined as

$$h_{0}(t, s)=1 $$

and

$$h_{k+1}(t,s)= \int_{s}^{t} h_{k}(\tau, s)\Delta\tau \quad\text{for all } s, t \in \mathbb{T}. $$

- When $\mathbb{T}=\mathbb{R}$, then for all $s, t \in\mathbb{T}$,
  $$h_{k}(t, s)=\frac{(t-s)^{k}}{k!}. $$
- When $\mathbb{T}=\mathbb{Z}$, then for all $s, t \in\mathbb{T}$,
  $$h_{k}(t, s)=\binom{t-s}{k}=\prod_{i=1}^{k} \frac{t-s+1-i}{i}. $$
- When $\mathbb{T}=q^{{\mathbb{N}}_{0}}$ with $q>1$, then for all $s, t \in\mathbb{T}$,
  $$h_{k}(t, s)=\frac{(t-s)^{k}_{q}}{[k]!} \quad\text{for } k\in{\mathbb{N}}_{0}, $$
  where $[k]_{q}:=\frac{q^{k}-1}{q-1}$ for $q\in\mathbb{R}\setminus \{1\}$ and $k\in{\mathbb{N}}_{0}$, $[k]!:= \prod_{j=1}^{k} [j]_{q}$ for $k\in{\mathbb{N}}_{0}$,
  $$(t-s)^{k}_{q}:= \prod_{j=0}^{k-1} \bigl(t-q^{j}s\bigr)\quad \text{for } k\in{\mathbb{N}}_{0}. $$

## Main results

In this section, we will state and prove two Ostrowski-Grüss type inequalities with a parameter *λ*. For this, we will need the following lemma which is given in [16, Lemma 1] but with some typos. We present here the correct version.

### Lemma 12

Generalized Montgomery identity with a parameter

*Suppose that*

1. $a, b\in\mathbb{T}$, $\lambda\in[0, 1]$, $I_{k}:a=x_{0}< x_{1}<\cdots <x_{k-1}<x_{k}=b$ *is a partition of the interval* $[a, b]$ *for* $x_{0}, x_{1}, \ldots,x_{k}\in\mathbb{T}$;
2. $\alpha_{j}\in\mathbb{T}$ ($j=0,1,\ldots, k+1$) *is* $k+2$ *points so that* $\alpha_{0}=a$, $\alpha_{j}\in[x_{j-1}, x_{j}]$ ($j=1,\ldots, k$) *and* $\alpha _{k+1}=b$;
3. $f: [a, b]\rightarrow\mathbb{R}$ *is a differentiable function*.

*Then we have the following equation*:

5 $$ \begin{aligned}[b] & \int_{a}^{b} K(t, I_{k})f^{\Delta}(t) \Delta t + \int_{a}^{b} f^{\sigma}(t)\Delta t \\ &\quad=(1-\lambda)\sum_{j=0}^{k} ( \alpha_{j+1}-\alpha_{j} )f(x_{j}) + \lambda\sum _{j=0}^{k} (\alpha_{j+1}- \alpha_{j} )\frac{f(\alpha _{j})+f(\alpha_{j+1})}{2}, \end{aligned}$$

*where*

6 $$ K(t, I_{k})= \textstyle\begin{cases} t- (\alpha_{1} -\lambda\frac{\alpha_{1}-a}{2} ), &t\in[a, \alpha_{1}),\\ t- (\alpha_{1} +\lambda\frac{\alpha_{2}-\alpha_{1}}{2} ), &t\in[\alpha _{1}, x_{1}),\\ t- (\alpha_{2} -\lambda\frac{\alpha_{2}-\alpha_{1}}{2} ), &t\in[x_{1}, \alpha_{1}),\\ \vdots&\\ t- (\alpha_{k-1} +\lambda\frac{\alpha_{k}-\alpha_{k-1}}{2} ),& t\in[\alpha_{k-1}, x_{k-1}),\\ t- (\alpha_{k} -\lambda\frac{\alpha_{k}-\alpha_{k-1}}{2} ),& t\in [x_{k-1}, \alpha_{k}),\\ t- (\alpha_{k} +\lambda\frac{\alpha_{k+1}-\alpha_{k}}{2} ),& t\in [\alpha_{k}, b]. \end{cases} $$

### Generalized Ostrowski-Grüss type inequality with a parameter I

We now state and prove our first result.

#### Theorem 13

*Suppose that*

1. $a, b\in\mathbb{T}$, $\lambda\in[0, 1]$, $I_{k}:a=x_{0}< x_{1}<\cdots <x_{k-1}<x_{k}=b$ *is a partition of the interval* $[a, b]$ *for* $x_{0}, x_{1}, \ldots,x_{k}\in\mathbb{T}$;
2. $\alpha_{j}\in\mathbb{T}$ ($j=0,1,\ldots, k+1$) *is* $k+2$ *points so that* $\alpha_{0}=a$, $\alpha_{j}\in[x_{j-1}, x_{j}]$ ($j=1,\ldots, k$) *and* $\alpha _{k+1}=b$;
3. $f: [a, b]\rightarrow\mathbb{R}$ *is differentiable*, $f^{\Delta}$ *is rd*-*continuous*, *and there exist* $\gamma, \Gamma\in\mathbb{R}$ *such that* $\gamma\leq f^{\Delta}(t)\leq\Gamma$ *for all* $t\in[a, b]$.

*Then we have the following inequality*:

7 $$\begin{aligned} & \Bigg|(1-\lambda)\sum_{j=0}^{k} (\alpha_{j+1}-\alpha_{j} )f(x_{j}) + \lambda\sum _{j=0}^{k} (\alpha_{j+1}- \alpha_{j} )\frac{f(\alpha _{j})+f(\alpha_{j+1})}{2}- \int_{a}^{b}f^{\sigma}(t)\Delta t \\ &\qquad{}- \frac{\Gamma+\gamma}{2} \sum_{j=0}^{k-1} \biggl[h_{2} \biggl(\alpha_{j+1}, \alpha_{j+1}- \lambda\frac{\alpha_{j+1}-\alpha_{j}}{2} \biggr) -h_{2} \biggl(x_{j}, \alpha_{j+1}-\lambda\frac{\alpha_{j+1}-\alpha_{j}}{2} \biggr) \biggr] \\ &\qquad{}- \frac{\Gamma+\gamma}{2} \sum_{j=0}^{k-1} \biggl[h_{2} \biggl(x_{j+1}, \alpha _{j+1}+\lambda \frac{\alpha_{j+2}-\alpha_{j+1}}{2} \biggr) -h_{2} \biggl(\alpha _{j+1}, \alpha_{j+1}+\lambda\frac{\alpha_{j+2}-\alpha_{j+1}}{2} \biggr) \biggr] \Bigg| \\ &\quad\leq\frac{\Gamma-\gamma}{2} \sum_{j=0}^{k-1} \biggl[h_{2} \biggl(x_{j}, \alpha _{j+1}-\lambda \frac{\alpha_{j+1}-\alpha_{j}}{2} \biggr)+h_{2} \biggl(\alpha _{j+1}, \alpha_{j+1}-\lambda\frac{\alpha_{j+1}-\alpha_{j}}{2} \biggr) \\ &\qquad{}+h_{2} \biggl(\alpha_{j+1}, \alpha_{j+1}+\lambda \frac{\alpha_{j+2}-\alpha _{j+1}}{2} \biggr) + h_{2} \biggl(x_{j+1}, \alpha_{j+1}+\lambda\frac{\alpha _{j+2}-\alpha_{j+1}}{2} \biggr) \biggr]. \end{aligned}$$

*Inequality* (7) *is sharp in the sense that the constant* 1/2 *on the right*-*hand side cannot be replaced by a smaller one*.

#### Proof

To proceed, we will need to make the following computations. For this, we apply the items of Theorem 10, where applicable, to get

8 $$ \begin{aligned}[b] \int_{a}^{b} K(t, I_{k})\Delta t ={}& \sum _{j=0}^{k-1} \biggl\{ \int_{x_{j}}^{\alpha _{j+1}} \biggl[t- \biggl(\alpha_{j+1}- \lambda\frac{\alpha_{j+1}-\alpha _{j}}{2} \biggr) \biggr]\Delta t \\ &+ \int_{\alpha_{j+1}}^{x_{j+1}} \biggl[t- \biggl(\alpha_{j+1}+ \lambda\frac {\alpha_{j+2}-\alpha_{j+1}}{2} \biggr) \biggr]\Delta t \biggr\} \\ ={}&\sum_{j=0}^{k-1} \biggl\{ \int_{x_{j}}^{\alpha_{j+1} - \lambda\frac{\alpha _{j+1}-\alpha_{j}}{2}} \biggl[t- \biggl(\alpha_{j+1}- \lambda\frac{\alpha _{j+1}-\alpha_{j}}{2} \biggr) \biggr]\Delta t \\ &+ \int_{\alpha_{j+1} - \lambda\frac{\alpha_{j+1}-\alpha_{j}}{2}}^{\alpha _{j+1}} \biggl[t- \biggl(\alpha_{j+1}- \lambda\frac{\alpha_{j+1}-\alpha _{j}}{2} \biggr) \biggr]\Delta t \\ &+ \int_{\alpha_{j+1}}^{\alpha_{j+1} +\lambda\frac{\alpha_{j+2}-\alpha _{j+1}}{2}} \biggl[t- \biggl(\alpha_{j+1}+ \lambda\frac{\alpha_{j+2}-\alpha _{j+1}}{2} \biggr) \biggr]\Delta t \\ &+ \int_{\alpha_{j+1} +\lambda\frac{\alpha_{j+2}-\alpha _{j+1}}{2}}^{x_{j+1}} \biggl[t- \biggl(\alpha_{j+1}+ \lambda\frac{\alpha _{j+2}-\alpha_{j+1}}{2} \biggr) \biggr]\Delta t \biggr\} \\ ={}&\sum_{j=0}^{k-1} \biggl\{ - \int_{\alpha_{j+1} - \lambda\frac{\alpha _{j+1}-\alpha_{j}}{2}}^{x_{j}} \biggl[t- \biggl(\alpha_{j+1}- \lambda\frac{\alpha _{j+1}-\alpha_{j}}{2} \biggr) \biggr]\Delta t \\ &+ \int_{\alpha_{j+1} - \lambda\frac{\alpha_{j+1}-\alpha_{j}}{2}}^{\alpha _{j+1}} \biggl[t- \biggl(\alpha_{j+1}- \lambda\frac{\alpha_{j+1}-\alpha _{j}}{2} \biggr) \biggr]\Delta t \\ &- \int_{\alpha_{j+1} +\lambda\frac{\alpha_{j+2}-\alpha _{j+1}}{2}}^{\alpha_{j+1}} \biggl[t- \biggl(\alpha_{j+1}+ \lambda\frac{\alpha _{j+2}-\alpha_{j+1}}{2} \biggr) \biggr]\Delta t \\ &+ \int_{\alpha_{j+1} +\lambda\frac{\alpha_{j+2}-\alpha _{j+1}}{2}}^{x_{j+1}} \biggl[t- \biggl(\alpha_{j+1}+ \lambda\frac{\alpha _{j+2}-\alpha_{j+1}}{2} \biggr) \biggr]\Delta t \biggr\} \\ ={}&\sum_{j=0}^{k-1} \biggl[h_{2} \biggl(\alpha_{j+1}, \alpha_{j+1}-\lambda \frac {\alpha_{j+1}-\alpha_{j}}{2} \biggr) -h_{2} \biggl(x_{j}, \alpha_{j+1}-\lambda\frac {\alpha_{j+1}-\alpha_{j}}{2} \biggr) \\ &+h_{2} \biggl(x_{j+1}, \alpha_{j+1}+\lambda \frac{\alpha_{j+2}-\alpha _{j+1}}{2} \biggr) -h_{2} \biggl(\alpha_{j+1}, \alpha_{j+1}+\lambda\frac{\alpha _{j+2}-\alpha_{j+1}}{2} \biggr) \biggr]. \end{aligned}$$

Following a similar approach, one gets

9 $$ \begin{aligned}[b] \int_{a}^{b}\big|K(t, I_{k})\big|\Delta t ={}& \sum _{j=0}^{k-1} \biggl[h_{2} \biggl(x_{j}, \alpha _{j+1}-\lambda\frac{\alpha_{j+1}-\alpha_{j}}{2} \biggr)+h_{2} \biggl(\alpha _{j+1}, \alpha_{j+1}- \lambda\frac{\alpha_{j+1}-\alpha_{j}}{2} \biggr) \\ &+h_{2} \biggl(\alpha_{j+1}, \alpha_{j+1}+\lambda \frac{\alpha_{j+2}-\alpha _{j+1}}{2} \biggr) + h_{2} \biggl(x_{j+1}, \alpha_{j+1}+\lambda\frac{\alpha _{j+2}-\alpha_{j+1}}{2} \biggr) \biggr]. \end{aligned} $$

Next we let $\Theta=\frac{\Gamma+\gamma}{2}$. From assumption 3, $\gamma\leq f^{\Delta}(t)\leq\Gamma$ for all $t\in[a,b]$ implies that $\gamma-\Theta\leq f^{\Delta}(t)-\Theta\leq\Gamma-\Theta$ for all $t\in [a,b]$. This further implies that $|f^{\Delta}(t)-\Theta|\leq\frac{\Gamma-\gamma}{2}$ for all $t\in [a,b]$. Hence,

10 $$ \max_{t\in[a,b]}\big|f^{\Delta}(t)-\Theta\big|\leq \frac{\Gamma-\gamma}{2}. $$

Using Lemma 12, we obtain

11 $$ \begin{aligned}[b] \int_{a}^{b} K(t, I_{k})f^{\Delta}(t) \Delta t ={}& (1-\lambda)\sum_{j=0}^{k} ( \alpha_{j+1}-\alpha_{j} )f(x_{j}) \\ &+ \lambda\sum_{j=0}^{k} ( \alpha_{j+1}-\alpha_{j} )\frac{f(\alpha _{j})+f(\alpha_{j+1})}{2} - \int_{a}^{b} f^{\sigma}(t)\Delta t. \end{aligned}$$

From relations (10) and (11), we get

12 $$ \bigg| \int_{a}^{b} K(t, I_{k}) \bigl(f^{\Delta}(t)-\Theta \bigr)\Delta t \bigg|\leq\frac{\Gamma-\gamma}{2} \int_{a}^{b} \big|K(t, I_{k})\big|\Delta t, $$

and

13 $$ \begin{aligned}[b] \int_{a}^{b} K(t, I_{k}) \bigl(f^{\Delta}(t)-\Theta \bigr)\Delta t ={}& (1-\lambda )\sum _{j=0}^{k} (\alpha_{j+1}- \alpha_{j} )f(x_{j}) \\ &+ \lambda\sum_{j=0}^{k} ( \alpha_{j+1}-\alpha_{j} )\frac{f(\alpha _{j})+f(\alpha_{j+1})}{2} - \int_{a}^{b} f^{\sigma}(t)\Delta t \\ &-\frac{\Gamma+\gamma}{2} \int_{a}^{b} K(t, I_{k})\Delta t. \end{aligned}$$

The desired inequality follows by using equations (8), (9), and (13) in inequality (12). □

#### Remark 14

By setting $\lambda=0$ in Theorem 13, we regain Theorem 3.

### Generalized Ostrowski-Grüss type inequality with a parameter II

Next, we present a generalization of Theorem 4 to *k* points.

#### Theorem 15

*Suppose that*

1. $a, b\in\mathbb{T}$, $\lambda\in[0, 1]$, $I_{k}:a=x_{0}< x_{1}<\cdots <x_{k-1}<x_{k}=b$ *is a partition of the interval* $[a, b]$ *for* $x_{0}, x_{1}, \ldots,x_{k}\in\mathbb{T}$;
2. $\alpha_{j}\in\mathbb{T}$ ($j=0,1,\ldots, k+1$) *is* $k+2$ *points so that* $\alpha_{0}=a$, $\alpha_{j}\in[x_{j-1}, x_{j}]$ ($j=1,\ldots, k$) *and* $\alpha _{k+1}=b$;
3. $f: [a, b]\rightarrow\mathbb{R}$ *is differentiable*.

*Then we have the following inequality*:

14 $$\begin{aligned} & \bigg|\frac{1-\lambda}{b-a}\sum _{j=0}^{k}(\alpha_{j+1}-\alpha _{j})f(x_{j})+ \frac{\lambda}{b-a}\sum _{j=0}^{k}(\alpha_{j+1}-\alpha _{j})\frac{f(\alpha_{j})+f(\alpha_{j+1})}{2} \\ & \qquad{}- \frac{1}{b-a} \int_{a}^{b}f^{\sigma}(t)\Delta t - \biggl( \frac{f(b)-f(a)}{(b-a)^{2}} \int_{a}^{b} K(t, I_{k})\Delta t \biggr) \bigg| \\ &\quad\leq \biggl[\frac{1}{b-a} \int_{a}^{b} K^{2}(t, I_{k}) \Delta t - \biggl(\frac{1}{b-a} \int_{a}^{b} K(t, I_{k})\Delta t \biggr)^{2} \biggr]^{\frac {1}{2}} \\ & \qquad{}\times \biggl[\frac{1}{b-a} \int_{a}^{b} \bigl(f^{\Delta}(t) \bigr)^{2}\Delta t - \biggl(\frac{f(b)-f(a)}{b-a} \biggr)^{2} \biggr]^{\frac{1}{2}}, \end{aligned}$$

*where* $K(t, I_{k})$ *is defined by* (6).

#### Proof

We start by making the following computations:

$$ \begin{gathered} \int_{a}^{b} \int_{a}^{b} \bigl(K(t, I_{k}) - K(s, I_{k}) \bigr) \bigl(f^{\Delta}(t) - f^{\Delta}(s) \bigr) \Delta t\Delta s \\ \quad = 2(b-a) \int_{a}^{b} K(t, I_{k})f^{\Delta}(t) \Delta t - 2 \biggl( \int_{a}^{b} K(t, I_{k})\Delta t \biggr) \biggl( \int_{a}^{b}f^{\Delta }(s)\Delta s \biggr). \end{gathered} $$

This implies that

15 $$ \begin{aligned}[b]&\frac{1}{b-a} \int_{a}^{b} K(t, I_{k})f^{\Delta}(t) \Delta t - \biggl(\frac{1}{b-a} \int_{a}^{b} K(t, I_{k})\Delta t \biggr) \biggl( \frac{1}{b-a} \int_{a}^{b}f^{\Delta}(s)\Delta s \biggr) \\ &\quad=\frac{1}{2(b-a)^{2}} \int_{a}^{b} \int_{a}^{b} \bigl(K(t, I_{k}) - K(s, I_{k}) \bigr) \bigl(f^{\Delta}(t) - f^{\Delta}(s) \bigr) \Delta t\Delta s. \end{aligned}$$

Following the same process, one gets the following identities:

16 $$ \begin{aligned}[b] &\frac{1}{b-a} \int_{a}^{b} K^{2}(t, I_{k}) \Delta t - \biggl(\frac{1}{b-a} \int_{a}^{b} K(t, I_{k})\Delta t \biggr)^{2} \\ &\quad=\frac{1}{2(b-a)^{2}} \int_{a}^{b} \int_{a}^{b} \bigl(K(t, I_{k}) - K(s, I_{k}) \bigr)^{2}\Delta t\Delta s \end{aligned}$$

and

17 $$ \begin{aligned}[b]&\frac{1}{b-a} \int_{a}^{b} \bigl(f^{\Delta}(t) \bigr)^{2}\Delta t - \biggl(\frac{1}{b-a} \int_{a}^{b} f^{\Delta}(t)\Delta t \biggr)^{2} \\ &\quad=\frac{1}{2(b-a)^{2}} \int_{a}^{b} \int_{a}^{b} \bigl(f^{\Delta}(t) - f^{\Delta}(s) \bigr)^{2}\Delta t\Delta s.\end{aligned} $$

From Lemma 12, we have

18 $$ \begin{aligned}[b]& \int_{a}^{b}K(t,I_{k})f^{\Delta}(t) \Delta t \\ &\quad=(1-\lambda)\sum_{j=0}^{k}( \alpha_{j+1}-\alpha_{j})f(x_{j}) +\lambda\sum _{j=0}^{k}(\alpha_{j+1}- \alpha_{j})\frac{f(\alpha_{j})+f(\alpha _{j+1})}{2}- \int_{a}^{b}f^{\sigma}(t)\Delta t.\end{aligned} $$

Using the Cauchy-Schwarz inequality on time scales, we get

19 $$ \begin{aligned}[b] &\bigg|\frac{1}{2(b-a)^{2}} \int_{a}^{b} \int_{a}^{b} \bigl(K(t, I_{k}) - K(s, I_{k}) \bigr) \bigl(f^{\Delta}(t) - f^{\Delta}(s) \bigr) \Delta t\Delta s \bigg| \\ &\quad\leq \biggl[\frac{1}{2(b-a)^{2}} \int_{a}^{b} \int_{a}^{b} \bigl(K(t, I_{k}) - K(s, I_{k}) \bigr)^{2}\Delta t\Delta s \biggr]^{\frac{1}{2}} \\ & \qquad{}\times \biggl[\frac{1}{2(b-a)^{2}} \int_{a}^{b} \int_{a}^{b} \bigl(f^{\Delta}(t) - f^{\Delta}(s) \bigr)^{2}\Delta t\Delta s \biggr]^{\frac{1}{2}}. \end{aligned} $$

Inequality (14) is achieved by applying (15)-(18) and the definition of definite integral (given in Section 2 above) to (19). □

#### Remark 16

Let $\lambda=0$ and $k=2$ in Theorem 15. If, in addition, we assume that $x\in[a, b]$, $\alpha_{0}=\alpha_{1}=x_{0}=a$; $\alpha_{2}=\alpha _{3}=x_{2}=b$; and $x_{1}=x$, then we recapture Theorem 4.

## Application to different time scales

In this section, we apply our theorems to different time scales to obtain completely new inequalities. We start with Theorem 13.

### Corollary 17

*Let* $\mathbb{T}=\mathbb{R}$. *Then we have*

20 $$\begin{aligned}[b] & \Bigg|(1-\lambda)\sum_{j=0}^{k} ( \alpha_{j+1}-\alpha_{j} )f(x_{j}) + \lambda\sum _{j=0}^{k} (\alpha_{j+1}- \alpha_{j} )\frac{f(\alpha _{j})+f(\alpha_{j+1})}{2}- \int_{a}^{b}f(t) \,\mathrm{d}t \\ &\qquad{}- \frac{\Gamma+\gamma}{2} \sum_{j=0}^{k-1} \biggl[\frac{\lambda^{2} (\alpha_{j+1}-\alpha_{j} )^{2}}{8} - \frac{ (2x_{j}- \lambda\alpha _{j}+(\lambda-2)\alpha_{j+1} )^{2}}{8} \biggr] \\ &\qquad{}- \frac{\Gamma+\gamma}{2} \sum_{j=0}^{k-1} \biggl[\frac{ (2x_{j+1}-\lambda\alpha_{j+2}+(\lambda-2)\alpha_{j+1} )^{2}}{8} - \frac {\lambda^{2} (\alpha_{j+2}-\alpha_{j+1} )^{2}}{8} \biggr]\Bigg| \\ &\quad\leq\frac{\Gamma-\gamma}{2} \sum_{j=0}^{k-1} \biggl[\frac{\lambda^{2} (\alpha_{j+1}-\alpha_{j} )^{2}}{8} + \frac{ (2x_{j}- \lambda\alpha _{j}+(\lambda-2)\alpha_{j+1} )^{2}}{8} \\ &\qquad{}+\frac{ (2x_{j+1}-\lambda\alpha_{j+2}+(\lambda-2)\alpha_{j+1} )^{2}}{8} + \frac{\lambda^{2} (\alpha_{j+2}-\alpha_{j+1} )^{2}}{8} \biggr].\end{aligned} $$

### Proof

The proof follows by applying Theorem 13 and using the facts that $f^{\sigma}(t)=f(t)$ and $h_{2}(t, s)=\frac{(t-s)^{2}}{2}$ (from the first item of Definition 11 for the case $k=2$). □

### Corollary 18

*Let* $\mathbb{T}=\mathbb{Z}$, $a=0$, *and* $b=n$. *Suppose also*

1. $\mathbb{I}_{k}:=\{j_{0}, j_{1},\ldots, j_{k}\}\subset\mathbb{Z}$, *where* $0=j_{0}< j_{1}<\cdots<j_{k}=n$, *is a partition of the set* $[0,n]\cap \mathbb{Z}$;
2. $\{\alpha_{0},\alpha_{1},\ldots,\alpha_{k}\}\subset\mathbb{Z}$ *is a set of* $k+2$ *points such that* $\alpha_{0}=0$, $\alpha_{i}\in[j_{i-1},j_{i}]$ *for* $i=1,2,\ldots,k$ *and* $\alpha_{k+1}=b$;
3. $f(k)=x_{k}$.

21 $$ \begin{aligned}[b] & \Bigg|(1-\lambda)\sum_{i=0}^{k} (\alpha_{i+1}-\alpha_{i} )x_{j_{i}} + \lambda\sum _{i=0}^{k} (\alpha_{i+1}- \alpha_{i} )\frac{x_{\alpha _{i}}+x_{\alpha_{i+1}}}{2}- \sum_{j=1}^{n}x_{j} \\ &\qquad{}- \frac{\Gamma+\gamma}{4} \Biggl[n^{2}-\sum _{j=0}^{k-1}(j_{i+1}-j_{i}) (2 \alpha_{i+1}+1-\lambda\alpha_{i+1}) \\ &\qquad{}+ \lambda \sum _{i=0}^{k-1}\alpha_{i+2}(\alpha_{i+1} - j_{i+1})+\lambda\sum_{i=0}^{k-1} \alpha_{i}(j_{i} -\alpha_{i+1}) \Biggr] \Bigg| \\ &\quad\leq\frac{\Gamma-\gamma}{4} \sum_{i=0}^{k-1} \biggl[j_{i} \bigl(j_{i}+(\lambda-2)\alpha_{i+1}- \lambda\alpha_{i}-1 \bigr)+ 2 \biggl(\lambda\frac {\alpha_{i+2}-\alpha_{i+1}}{2}+ \frac{1}{2} \biggr)^{2}-1 \\ &\qquad{}+j_{i+1} \bigl(j_{i+1} +(\lambda-2) \alpha_{i+1}-\lambda\alpha_{i+2}-1 \bigr) + 2 \biggl(\lambda \frac {\alpha_{i+1}-\alpha_{i}}{2}-\frac{1}{2} \biggr)^{2} \\ &\qquad{}+2\alpha_{i+1} \biggl((1-\lambda)\alpha_{i+1}+\lambda \frac{\alpha _{i+1}+\alpha_{i}}{2}+1 \biggr) \biggr]. \end{aligned}$$

### Proof

The intended inequality follows by applying Theorem 13 and the fact that $h_{2}(t,s)=\frac{(t-s)(t-s-1)}{2}$. □

### Remark 19

By setting $\lambda=0$ in Corollaries 17 and 21, we recover Corollaries 23 and 25 of the paper [14]. For $\lambda\in (0, 1]$, we get new inequalities.

Next, we turn our attention to Theorem 15.

### Corollary 20

*If* $\mathbb{T}=\mathbb{R}$, *then the inequality in Theorem* 15 *becomes*

22 $$ \begin{aligned}[b] & \Bigg|\frac{1-\lambda}{b-a}\sum _{i=0}^{k}(\alpha_{i+1}-\alpha _{i})f(x_{i})+ \frac{\lambda}{b-a}\sum _{i=0}^{k}(\alpha_{i+1}-\alpha _{i})\frac{f(\alpha_{i})+f(\alpha_{i+1})}{2} \\ & \qquad{}- \frac{1}{b-a} \int_{a}^{b}f(t)\,\mathrm{d}t - \biggl(\frac{f(b)-f(a)}{(b-a)^{2}} \int_{a}^{b} K(t, I_{k})\,\mathrm{d}t \biggr) \Bigg| \\ &\quad\leq \biggl[\frac{1}{b-a} \int_{a}^{b} K^{2}(t, I_{k})\,\mathrm{d}t - \biggl(\frac{1}{b-a} \int_{a}^{b} K(t, I_{k})\,\mathrm{d}t \biggr)^{2} \biggr]^{\frac{1}{2}} \\ &\qquad{} \times \biggl[\frac{1}{b-a} \int_{a}^{b} \bigl(f'(t) \bigr)^{2}\,\mathrm{d}t - \biggl(\frac{f(b)-f(a)}{b-a} \biggr)^{2} \biggr]^{\frac{1}{2}}. \end{aligned} $$

### Corollary 21

*Let* $\mathbb{T}=\mathbb{Z}$, $a=0$, *and* $b=n$. *Suppose also*

1. $\mathbb{I}_{k}:=\{j_{0}, j_{1},\ldots, j_{k}\}\subset\mathbb{Z}$, *where* $0=j_{0}< j_{1}<\cdots<j_{k}=n$, *is a partition of the set* $[0,n]\cap \mathbb{Z}$;
2. $\{\alpha_{0},\alpha_{1},\ldots,\alpha_{k}\}\subset\mathbb{Z}$ *is a set of* $k+2$ *points such that* $\alpha_{0}=0$, $\alpha_{i}\in[j_{i-1},j_{i}]$ *for* $i=1,2,\ldots,k$ *and* $\alpha_{k+1}=b$;
3. $f(k)=x_{k}$.

*We have the inequality*

23 $$\begin{aligned} & \Bigg|\frac{1-\lambda}{n}\sum _{i=0}^{k}(\alpha_{i+1}- \alpha_{i})x_{j_{i}}+ \frac{\lambda}{n}\sum _{i=0}^{k}(\alpha_{i+1}- \alpha_{i})\frac{x_{\alpha _{i}}+x_{\alpha_{i+1}}}{2} \\ & \qquad{}- \frac{1}{n}\sum_{j=1}^{n}x_{j} - \Biggl(\frac{x_{n}-x_{0}}{n^{2}}\sum_{j=0}^{n-1} K(j, I_{k}) \Biggr) \Bigg| \\ &\quad\leq \Biggl[\frac{1}{n}\sum_{j=0}^{n-1} K^{2}(j, I_{k}) - \Biggl(\frac{1}{n}\sum _{j=0}^{n-1} K(j, I_{k}) \Biggr)^{2} \Biggr]^{\frac {1}{2}} \\ & \qquad{}\times \Biggl[\frac{1}{n}\sum_{j=0}^{n-1} (x_{j+1}-x_{j} )^{2} - \biggl(\frac{x_{n}-x_{0}}{n} \biggr)^{2} \Biggr]^{\frac{1}{2}}. \end{aligned}$$

### Corollary 22

*Let* $\mathbb{T}=q^{\mathbb{N}_{0}}$, $q>1$, $a=q^{m}$, $b=q^{n}$ *with* $m, n\in \mathbb{N}$ *and* $m< n$. *Suppose that*

1. $\mathbb{I}_{k}:q^{m}=q^{j_{0}}< q^{j_{1}}<\cdots<q^{j_{k}}=q^{n}$ *is a partition of the set* $[q^{m},q^{n}]\cap q^{\mathbb{N}_{0}}$ *for* $j_{0},j_{1},\ldots,j_{k}\in{\mathbb{N}_{0}}$;
2. $q^{\alpha_{i}}\in q^{\mathbb{N}_{0}}$ ($i=0,1,\ldots,k+1$) *is a set of* $k+2$ *points such that* $q^{\alpha_{0}}=q^{m}$, $q^{\alpha_{i}}\in [q^{j_{i-1}},q^{j_{i}}]\cap q^{\mathbb{N}_{0}}$ ($i=1,2,\ldots,k$) *and* $q^{\alpha_{k+1}}=q^{n}$;
3. $f:[q^{m},q^{n}]\to\mathbb{R}$ *is differentiable*.

*Then we have the inequality*

24 $$ \begin{aligned}[b] & \Bigg|\frac{1-\lambda}{q^{n}-q^{m}}\sum _{i=0}^{k}\bigl(q^{\alpha _{i+1}}-q^{\alpha_{i}} \bigr)f\bigl(q^{j_{i}}\bigr)+ \frac{\lambda}{q^{n}-q^{m}}\sum _{i=0}^{k}\bigl(q^{\alpha_{i+1}}-q^{\alpha_{i}} \bigr)\frac{f(q^{\alpha _{i}})+f(q^{\alpha_{i+1}})}{2} \\ & \qquad{}- \frac{1}{q^{n}-q^{n}} \int_{q^{m}}^{q^{n}}f(qt)\,\mathrm{d}_{q}t - \biggl( \frac{f(q^{n})-f(q^{m})}{(q^{n}-q^{m})^{2}} \int_{q^{m}}^{q^{n}} K(t, I_{k})\,\mathrm{d}_{q}t \biggr)\Bigg| \\ &\quad\leq \biggl[\frac{1}{q^{n}-q^{m}} \int_{q^{m}}^{q^{n}} K^{2}(t, I_{k})\,\mathrm{d}_{q}t - \biggl(\frac{1}{q^{n}-q^{m}} \int_{q^{m}}^{q^{n}} K(t, I_{k})\,\mathrm{d}_{q}t \biggr)^{2} \biggr]^{\frac{1}{2}} \\ & \qquad{}\times \biggl[\frac{1}{q^{n}-q^{m}} \int_{q^{m}}^{q^{n}} \biggl(\frac {f(qt)-f(t)}{(q-1)t} \biggr)^{2}\,\mathrm{d}_{q}t - \biggl(\frac{f(q^{n})-f(q^{m})}{q^{n}-q^{m}} \biggr)^{2} \biggr]^{\frac{1}{2}}. \end{aligned} $$

## Conclusion

In this paper, we proved two Ostrowski-Grüss type inequalities for *k* points on time scales. Our results contain a parameter *λ* such that for $\lambda=0$, our first theorem boils down to Theorem 3. The second theorem extends and generalizes Theorem 4 to *k* points. By choosing different values of *λ* in $[0, 1]$ and $k\in\mathbb{N}$, one can generate loads of interesting new inequalities that can come handy in approximation or numerical analysis. Finally, we applied our results to the continuous, discrete, and quantum time scales to obtain more results in this direction.
